# 

**Published:** 1883-04

**Authors:** 


### Pamphlets.

---

The Best Methods of Treating Operative Wounds. By Henry O. Marcy, A.M., M.D., Boston. Reprint from *Medical Gazette*.

Annual Address delivered before the American Academy of Medicine at Philadelphia, October 26, 1882. By Traill Green, A.M., M.D., President.

Preventable Disease Circulars. Typhoid fever, scarlet fever, diphtheria. Illinois State Board of Health.

Addresses delivered on the occasion of the Dedication of Cooper Medical College Building, San Francisco. By Levi C. Lane, M.D., and Edward R. Taylor.

Third Biennial Report of the Trustees, Superintendent, Treasurer and Architect of the Illinois Eastern Hospital for the Insane, at Kankakee.

Fifth Biennial Report of the Trustees, Superintendent and Treasurer of the Illinois Southern Hospital for the Insane, at Anna.

